# Hidden in Plain Sight: The Diagnostic Odyssey of Glomus Tumors

**DOI:** 10.7759/cureus.115832

**Published:** 2026-09-05

**Authors:** Elizabeth Kern, Olivia C Stanley, Otto Fisher, Gary Schwartz

**Affiliations:** 1 Orthopedic Surgery, Nova Southeastern University Dr. Kiran C. Patel College of Allopathic Medicine, Fort Lauderdale, USA; 2 Medicine, Nova Southeastern University Dr. Kiran C. Patel College of Allopathic Medicine, Fort Lauderdale, USA; 3 Medical Education, Nova Southeastern University Dr. Kiran C. Patel College of Allopathic Medicine, Fort Lauderdale, USA

**Keywords:** diagnostic delay, glomus tumor, hand surgery, satellite lesion, tumor recurrence

## Abstract

Glomus tumors are benign mesenchymal neoplasms arising from specialized neurovascular dermal structures known as glomus bodies, which participate in thermoregulation by modulating cutaneous blood flow. While these tumors are predominantly located in the subungual and periungual regions of the distal extremities, they may arise at extradigital sites, where atypical presentations frequently delay diagnosis. Despite the availability of a well-established clinical triad, these tumors are notoriously difficult to diagnose promptly.

We present three surgically confirmed cases of glomus tumor, each representing a distinct clinical presentation: a classic subungual digital tumor, an extradigital forearm tumor, and a recurrent digital tumor. All three cases were characterized by significant diagnostic delay. A comprehensive literature review was conducted using PubMed, with search terms including "glomus tumor", "diagnostic delay", "recurrence", "extradigital glomus tumor", and "satellite lesion". The search included articles published between 1972 and 2026. Relevant case reports, retrospective series, and review articles were reviewed and synthesized in conjunction with the presented cases.

The three cases illustrate that diagnostic delay in glomus tumors is a widespread and recurring pattern with significant clinical consequences. Delayed diagnosis deprives patients of timely appropriate preoperative imaging, compromises surgical planning, and may increase the risk through the failure to identify satellite lesions preoperatively. Early application of the bedside diagnostic triad, combined with routine preoperative imaging, is essential to improving outcomes for this frequently overlooked tumor. The three cases presented share several common clinical features. Histopathologic diagnosis was similar in all three cases. The pain and tenderness on physical examination was a prominent clinical feature of all the cases. They all presented a diagnostic challenge as well as a delayed diagnosis. Surgical excision of all three lesions resulted in complete resolution of the symptoms and signs. The significance of the common findings is that even with a recurrent lesion, as well as an extra-digital location, glomus tumors can have various presentations, but the underlying features are recognizable.

## Introduction

Glomus bodies are minute neurovascular structures located in the dermis that play an important role in thermoregulation. They are found in high concentrations in ears, palms, fingertip pads, and nail beds [1]. More specifically, glomus bodies contain arteriovenous shunts that modulate cutaneous blood flow and are enclosed within connective tissue capsules [2]. These structures, while often overlooked, are vital to maintaining homeostasis.

There are three well-known histological subtypes: solid glomus tumor, glomangioma, and glomangiomyoma. The typical solid glomus tumor consists of uniformly round, small glomus cells with pale eosinophilic cytoplasm and relatively few vascular or smooth muscle cells. Glomangiomas are primarily composed of dilated, branching vascular channels mixed with glomus cells. They usually present as multiple lesions. Glomangiomas are primarily composed of dilated, branching vascular channels mixed with glomus cells, and glomangiomyomas are a rarer variant that combines all three components into one [2]. The differentiating factor is the presence of smooth muscle cells. These tumors are found primarily in the distal extremities; 50-75% occur in the hand, and 89% of hand lesions are located at the level of the distal phalanx [3].

Glomus tumors are united by a significant diagnostic delay. Whether prolonged delay contributes to higher rates of tumor recurrence - potentially through the undetected growth of synchronous satellite lesions - remains an underexplored question. Although existing work has documented recurrence rates as high as 12-33% and proposed satellite lesions as their likely mechanism, to date, no study has directly examined whether the length of time to diagnosis influences the likelihood of glomus tumor recurrence [4-6]. Nevertheless, the literature has suggested a potential association between delayed diagnosis and recurrence, with prolonged diagnostic intervals allowing otherwise occult or synchronous lesions to persist and later present as recurrent disease. This proposed relationship provides a rationale for examining diagnostic delay as a potential contributor to recurrence rather than viewing recurrence solely as a consequence of incomplete surgical excision [4-6]. This represents an important gap in the current literature. If prolonged diagnostic delay permits satellite lesions to mature and become symptomatic following initial surgery, earlier diagnosis - facilitated by routine application of the established clinical triad and comprehensive preoperative imaging - may reduce recurrence burden. This recurrence burden may reflect delayed diagnosis as much as surgical technique. We hypothesize that diagnostic delay is an independent contributor to glomus tumor recurrence through the mechanism of undetected synchronous satellite lesion growth.

Through a comprehensive review of the literature and detailed presentation of these cases, we investigate the relationship between delayed diagnosis and recurrence and advocate for heightened clinical awareness to improve outcomes for this frequently overlooked tumor. The cases presented in this report were evaluated retrospectively. The cases were identified and treated by the senior author between 2008 and 2012. These cases were not consecutive patients because they were selected based on their interesting variations.

This study was conducted for educational purposes only and did not involve identifiable patient information. Informed consent for treatment and open access publication was obtained or waived by all participants in this study. Institutional review board (IRB) approval was not required, nor was it obtained.

## Case presentation

Case 1

A 42-year-old right-hand-dominant man presented with a chief complaint of pain in the right thumb for three years. There was no history of trauma. The pain had increased over the years. Three years after the pain started, the patient presented to an orthopedic surgeon and, one year later, to another orthopedic surgeon. He was treated for unspecified hand pain with various splints to relieve pressure on the painful area. One year later, he presented to a hand surgeon. The pain was located on the ulnar aspect of the paronychial region of the thumb, just proximal to the nail plate. The patient noticed a bluish discoloration under the nail plate. The pain was described as sharp, especially when the painful area was palpated. There was an area of exquisite tenderness just proximal to the ulnar base of the nail. He experienced cold sensitivity.

On examination of the right upper extremity, there were no abnormalities of the shoulder, elbow, forearm, or wrist. On examination of the right thumb, there was a bluish discoloration along the base of the nail plate. There was an area of exquisite tenderness just proximal to the ulnar base of the nail plate, where the eponychium met the ulnar paronychium. There was no tenderness in the remaining areas of the nail (Figure 1).

**Figure 1:**
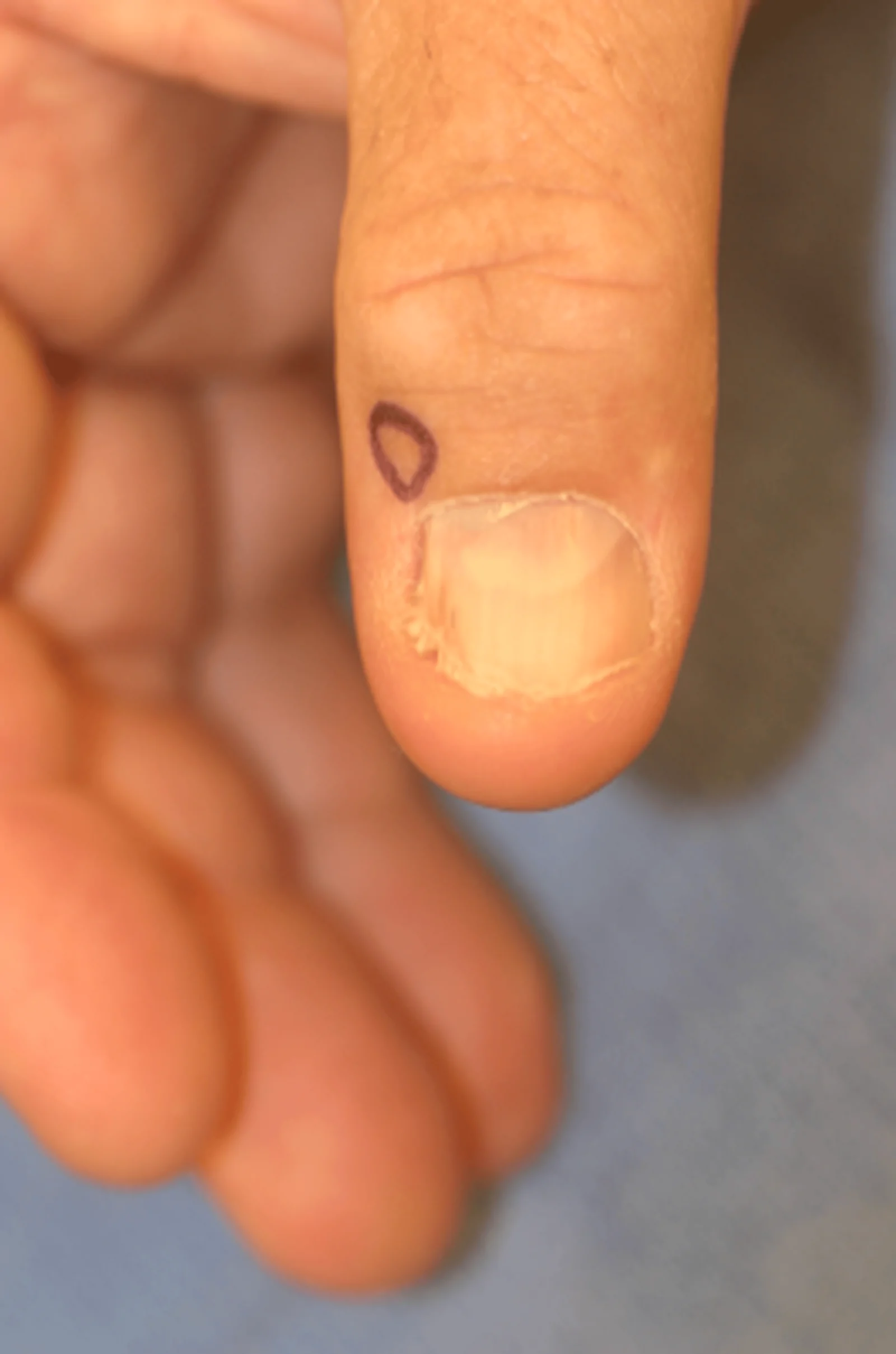
Preoperative photograph of the thumb demonstrating the location of the pain (purple circle).

Radiographs of the right thumb did not demonstrate any abnormalities. Magnetic resonance imaging (MRI) of the right thumb demonstrated a 7 x 6 x 4 mm (CC x AP x TRV) hyperintense lesion along the dorsoulnar aspect of the distal phalanx of the thumb, beneath the nail. No underlying marrow signal abnormality was seen, and no osseous erosion was identified (Figure 2).

**Figure 2:**
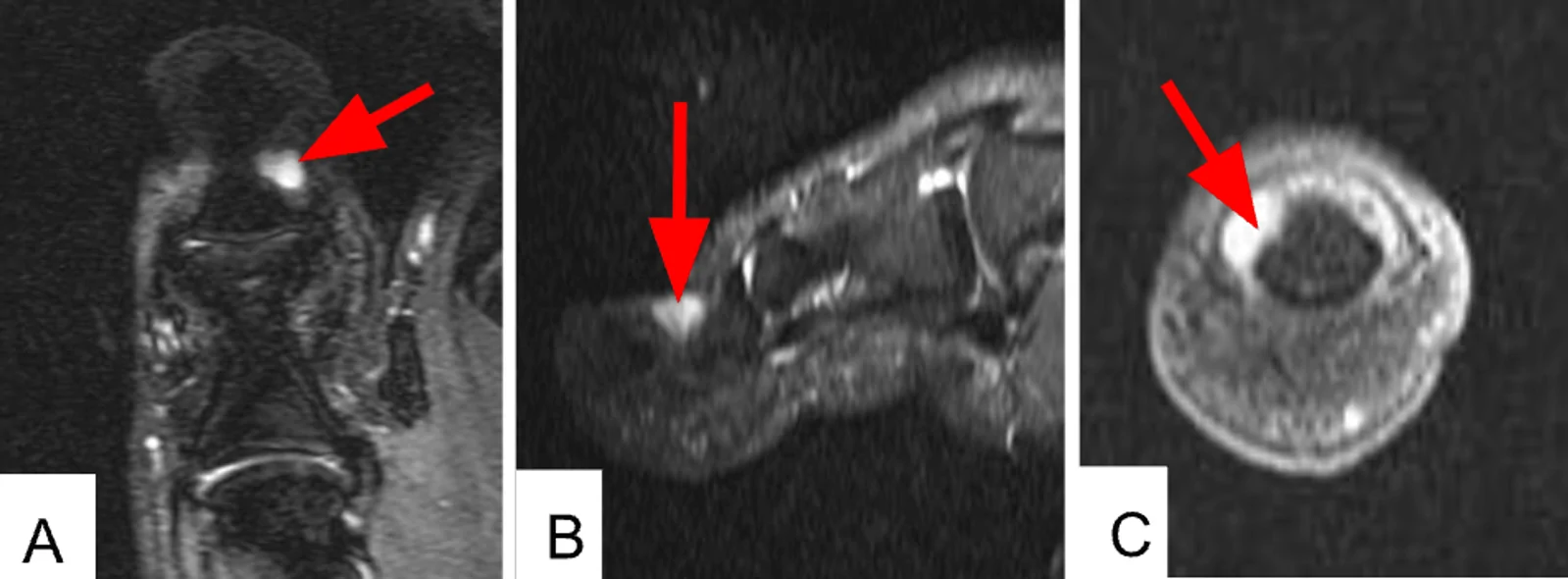
Preoperative MRI of the right thumb Preoperative (A) coronal, (B) sagittal, and (C) axial MRI of the right thumb demonstrates the enhancing lesion on the dorsoulnar aspect of the distal phalanx of the thumb (red arrows). MRI, magnetic resonance imaging

The patient was taken to the operating room, where the subungual mass was excised. An incision was made along the base of the interval between the eponychial fold and the ulnar paronychial region of the right thumb. The ulnar half of the nail plate was removed. A purplish-colored soft tissue mass was identified in the region of the germinal matrix. The mass was circumferentially dissected and excised (Figure 3).

**Figure 3:**
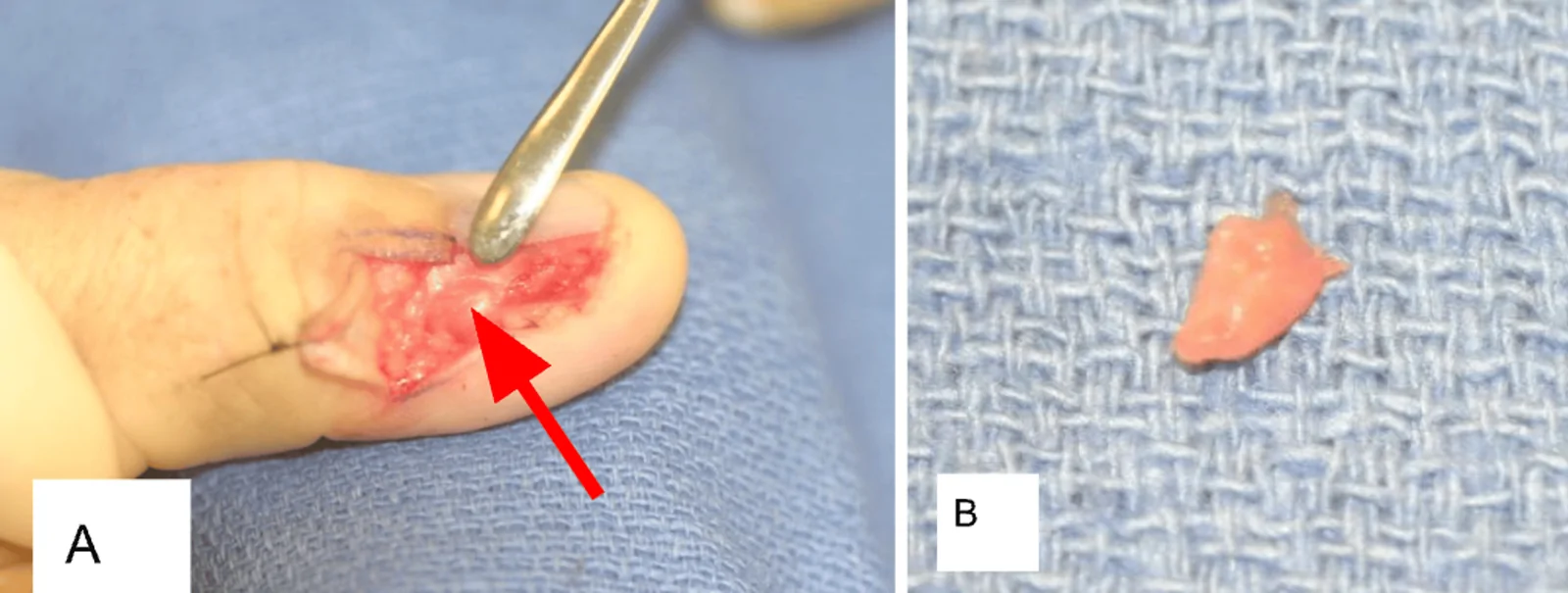
Intraoperative photographs Intraoperative view (A) of the mass located in the proximal aspect of the nail bed and (B) the specimen once it was removed.

The histopathologic examination demonstrated cells that were positive for smooth muscle actin and CD34 and were negative for S-100 protein, desmin, and pan-keratin (AE1/AE3/PCK26), supporting the diagnosis of a glomus tumor (Figure 4).

**Figure 4:**
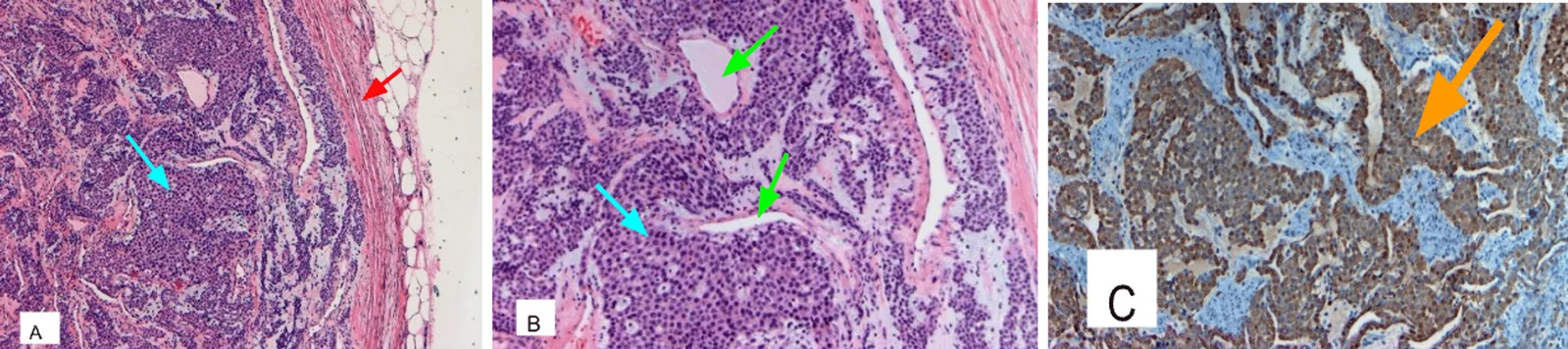
Histopathology of the tumor Histopathology of the mass with H&E stain under low power (x10) (A) and high power (x20) (B) demonstrates the tumor capsule (red arrow), the tumor cells (blue arrows), and the blood vessels (green arrows). SMA stain (x10, C) demonstrates the brown pigment representing the positive staining of the tumor cells (orange arrow). H&E, hematoxylin and eosin; SMA, smooth muscle actin

The patient was seen at 1 week, 2 weeks, 5 weeks, and 13 weeks postoperatively. There was no pain or tenderness. Full composite active motion of all digits was observed. He was discharged from active care at that time. The clinical timeline is presented in Table 1.

**Table 1:** The clinical timeline of the patient’s care The table summarizes the clinical course of treatment from the time of symptom presentation to discharge from active care.

Timeline (Months)	Event
0	First noticed symptoms
1-48	Seen by multiple physicians and the first orthopedic surgeon
50	Seen by a second orthopedic surgeon
64	Seen by a hand surgeon
68	Surgery by a hand surgeon
71	Discharged by a hand surgeon

Case 2 

A 52-year-old right-hand-dominant woman presented with pain in the subungual area of the right ring finger for 20 years. She had a mass removed from the right ring finger in 1990 and then again in 1992 due to a recurrence. She was told the mass was a glomus tumor. The pain had progressively increased over the preceding two decades. She reported cold intolerance of the ring finger.

On physical examination of the right upper extremity, there were no abnormalities of the shoulder, elbow, forearm, or wrist. On examination of the right ring finger, there was tenderness along the nail plate. There was a mild bluish discoloration at the base of the nail plate. There was exquisite tenderness to palpation over the nail plate.

Radiographs demonstrated a mild bony concavity on the dorsal aspect of the distal phalanx (Figure 5).

**Figure 5:**
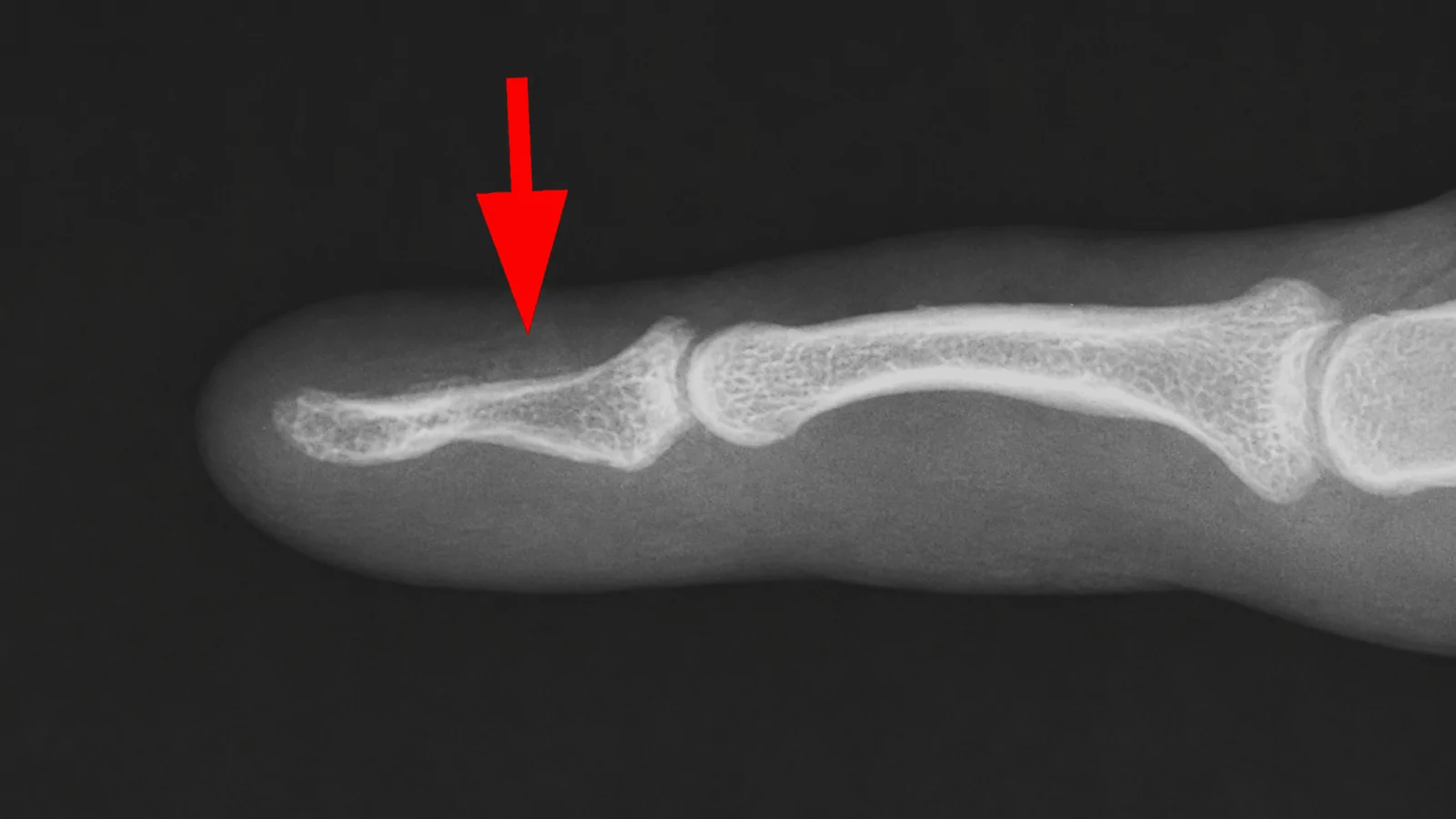
Preoperative photograph of the ring finger demonstrating a concavity on the dorsal aspect of the distal phalanx (red arrow).

MRI demonstrated soft tissue thickening along the dorsal aspect of the distal phalanx of the right ring finger, with questionable erosive changes suspicious for recurrent glomus tumor (Figure 6).

**Figure 6:**
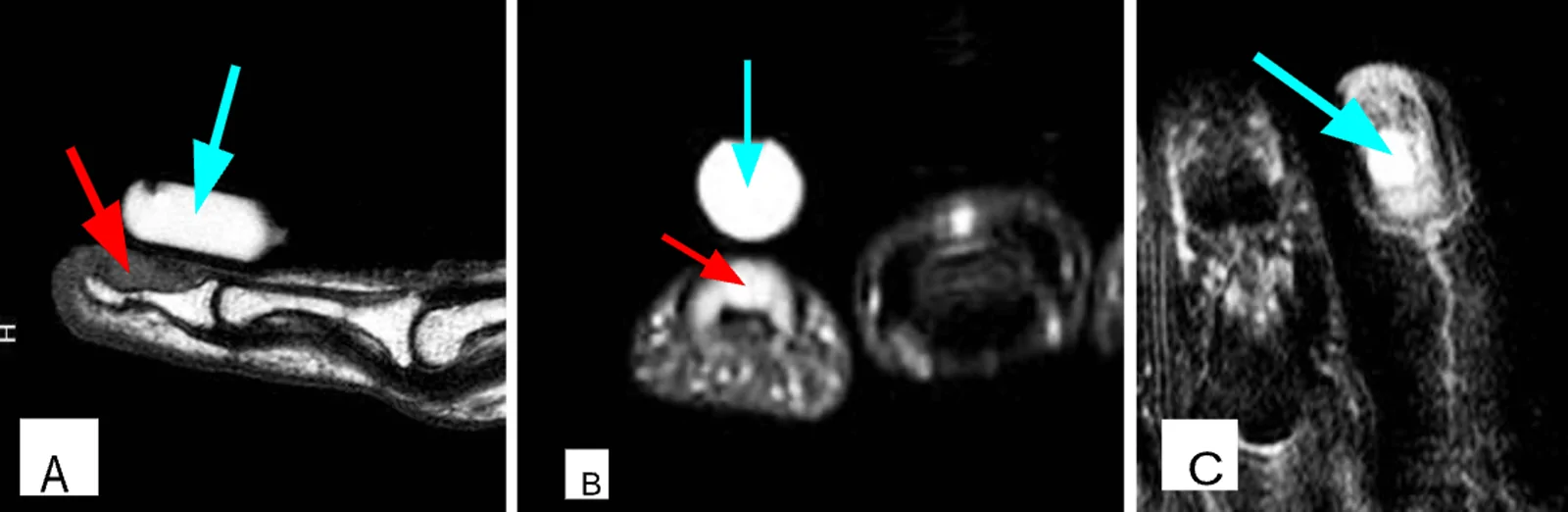
Preoperative MRI of the ring finger (A) Sagittal, (B) axial, and (C) coronal MRI images of the right ring finger demonstrating abnormal soft tissue thickening (red arrows) in the dorsal aspect of the distal phalanx, and the vitamin capsule for identification purposes (blue arrows). MRI, magnetic resonance imaging

The patient was taken to the operating room for the excision of a subungual mass. The nail plate was elevated from the nail bed. Some bogginess was noted at the junction of the sterile and germinal matrices. A longitudinal incision was made in the nail bed. A soft tissue mass extending to the dorsal aspect of the distal phalanx was identified (Figure 7).

**Figure 7:**
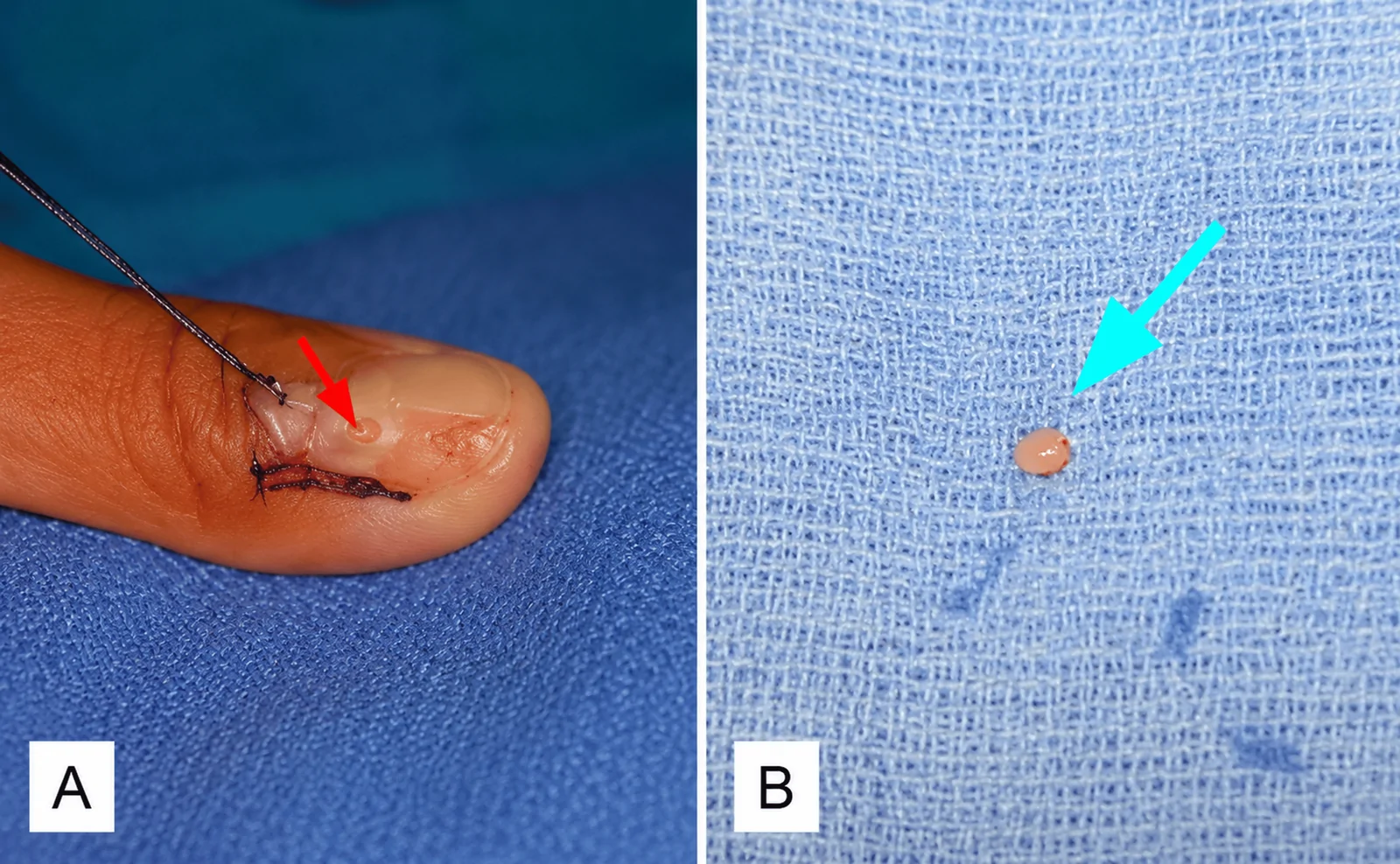
(A) Intraoperative photograph showing the tumor identified at the junction of the germinal and sterile matrix of the nail bed (red arrow), and (B) the tumor once it was excised (blue arrow).

Histopathologic examination demonstrated positivity for smooth muscle actin (SMA) and negativity for pancytokeratin. The findings were consistent with a glomus tumor (Figure 8).

**Figure 8:**
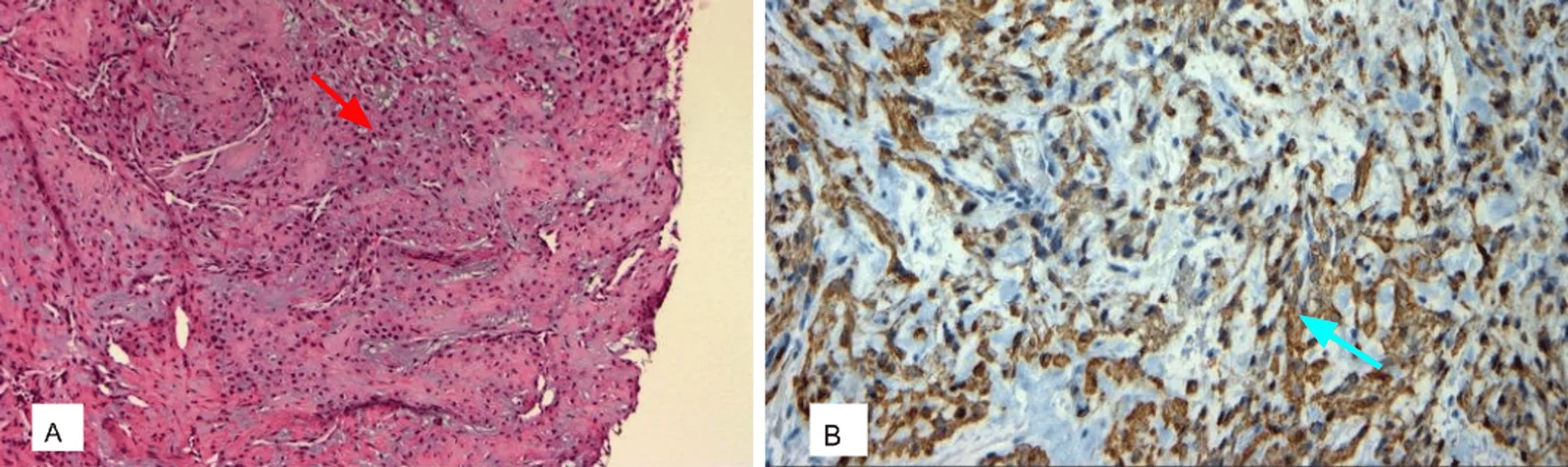
Histopathology of the tumor Histopathology of the mass with H&E stain under low power (x10) demonstrating (A) the bluish cells, which are representative of the tumor cells (red arrow), and (B) under high power (x40) SMA staining demonstrating the positivity for SMA (blue arrow). H&E, hematoxylin and eosin; SMA, smooth muscle actin

The patient was seen at one, two, and five weeks postoperatively. She reported no residual pain, and there was no tenderness on examination. There was full composite motion of the digits. The patient was discharged from active care at that time. The clinical timeline is presented in Table 2.

**Table 2:** Timeline of the patient’s clinical course

Timeline (Months)	Event
0	First noticed symptoms
60	First surgery by a hand surgeon
84	Second surgery by a hand surgeon
240	Third surgery by a hand surgeon
242	Discharged by a hand surgeon

Case 3

A 46-year-old right-hand-dominant man presented with a chief complaint of a painful mass on the right forearm for 10 years. The pain had progressively increased, particularly in the year prior to presentation. There was no history of trauma. The mass increased in size over the years. The patient stated that whenever the mass was touched, he had exquisite tenderness associated with a shock-like sensation. The patient also reported cold sensitivity associated with the mass. He was evaluated by multiple physicians over the years, including a psychiatrist, as none of the other physicians had been able to determine the etiology of his symptoms.

On physical examination of the right upper extremity, there were no abnormal findings in the shoulder or elbow. There was a 5 x 5 mm bluish-appearing soft tissue mass on the volar radial aspect of the distal third of the forearm (Figure 9). The mass was extremely tender to palpation. Capillary refill was less than 2 seconds. Skin turgor was normal.

**Figure 9:**
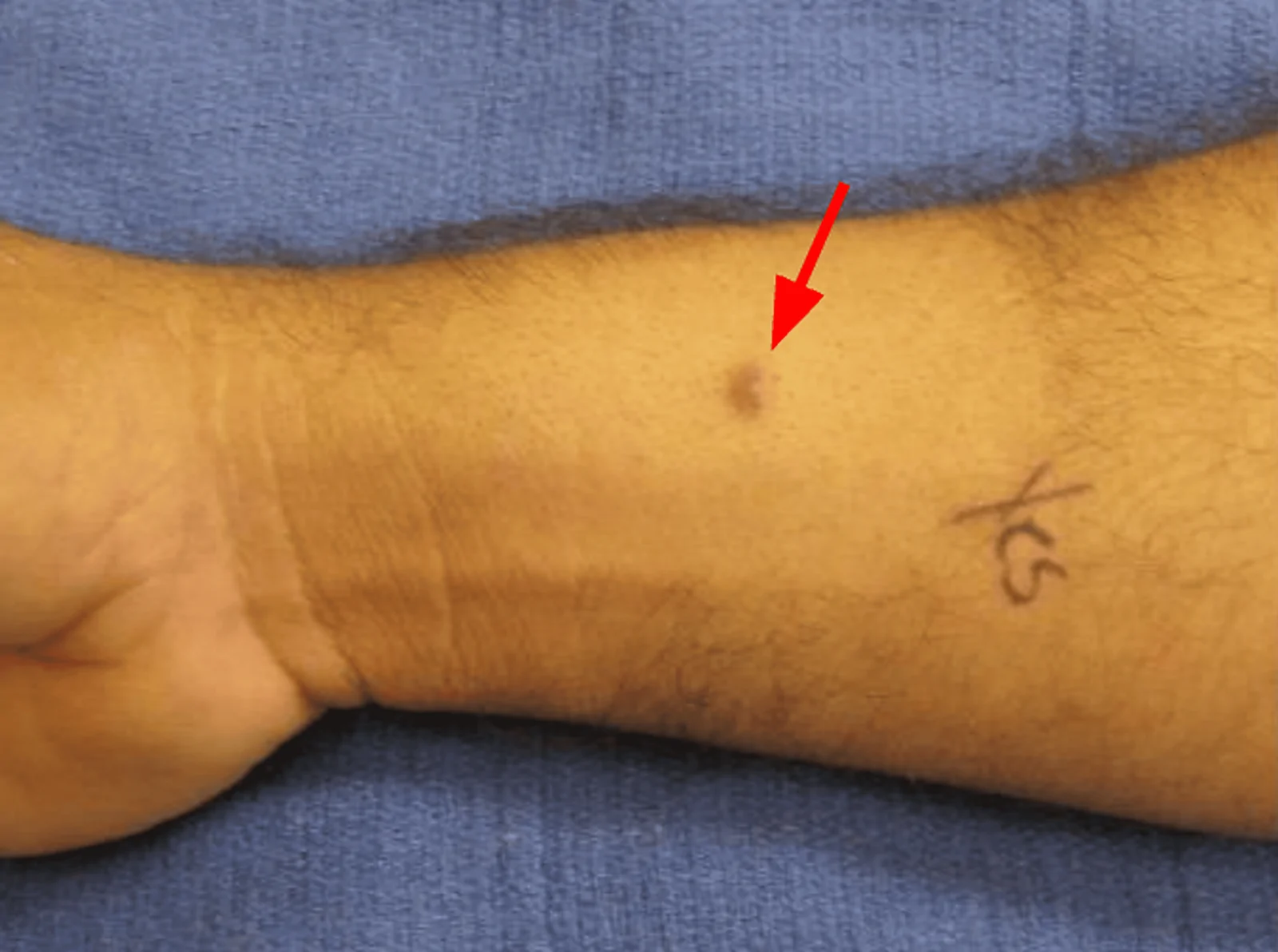
Preoperative appearance of the purplish colored mass on the volar surface of the right distal forearm.

X-rays of the right distal forearm did not demonstrate any abnormalities. The patient was taken to the operating room, where the mass was excised. An elliptical incision was made surrounding the soft tissue mass. The soft tissue mass was bluntly dissected and excised (Figure 10).

**Figure 10:**
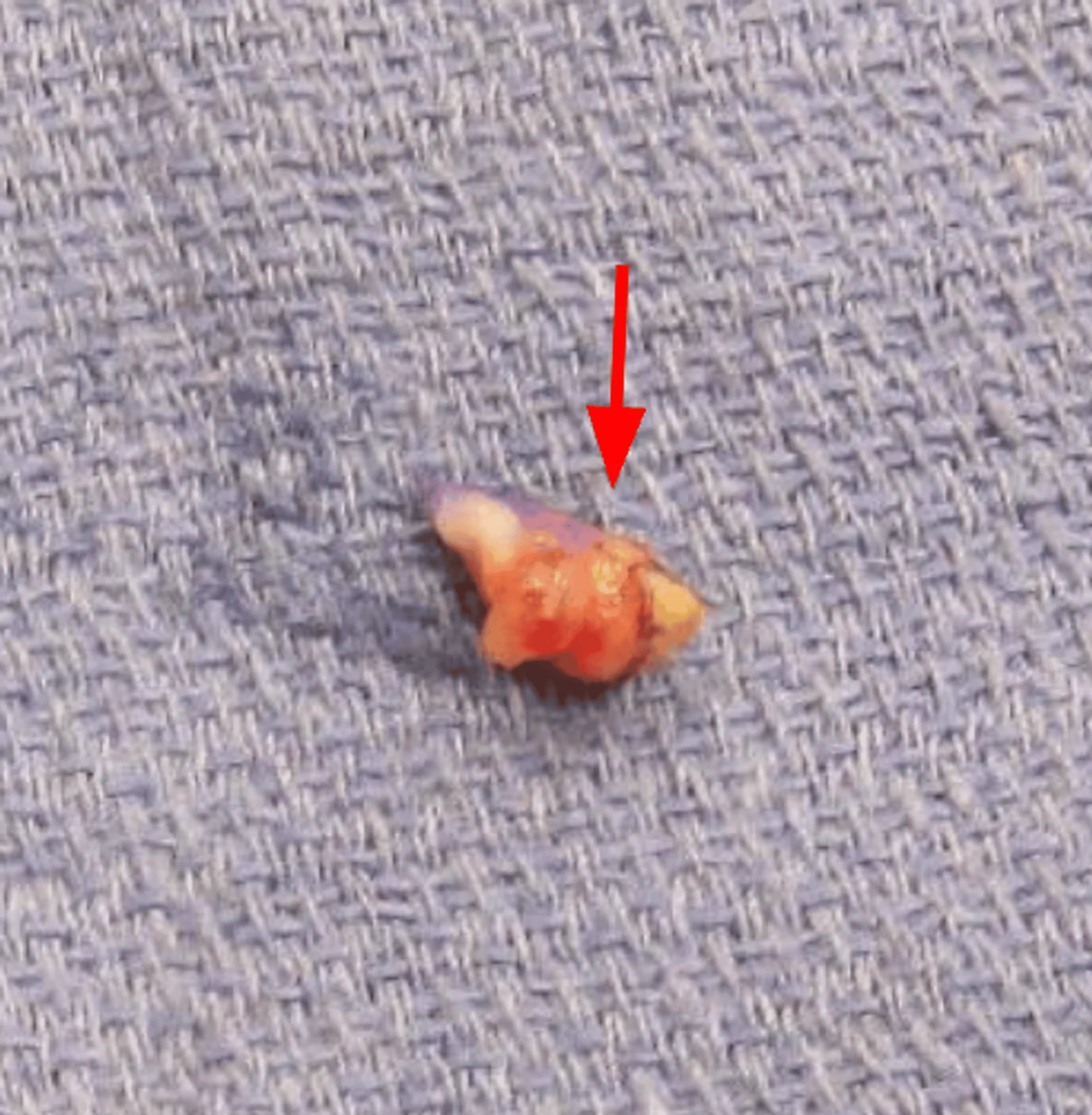
Specimen excised The specimen removed consisted of a 0.4-cm grey-tan lesion (red arrow).

The histopathology demonstrated a tumor that was positive for SMA and CD34 (focal). It demonstrated less than 1% positivity for Ki-67. S-100 protein, desmin, pan-keratin (AE1/AE3/PCK26), and CK 8/18 were negative. These findings supported the diagnosis of a glomus tumor (Figure 11).

**Figure 11:**
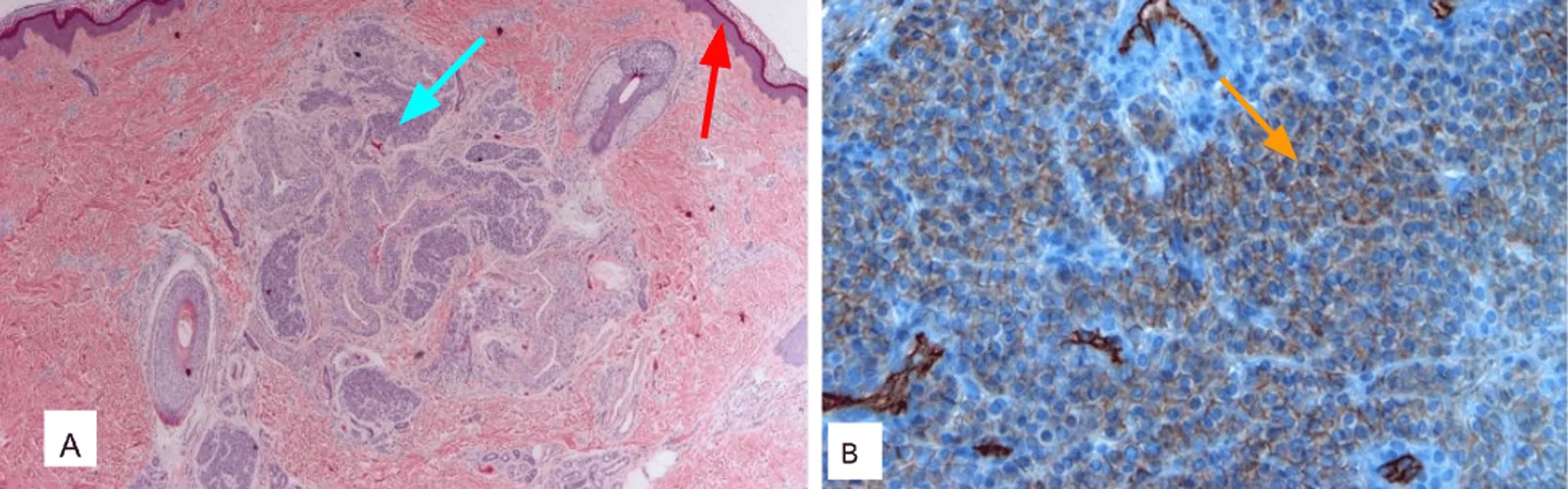
Histopathology of the tumor Histopathology of the mass with H&E stain under low power (x2.5), demonstrating (A) epidermis (red arrow) and the tumor cells (blue arrow), and (B) CD34 IHC stain demonstrating the tumor cells (orange arrow). H&E, hematoxylin and eosin; CD, cluster of differentiation; IHC, immunohistochemistry

The patient was seen at one, two, and five weeks postoperatively. At the final follow-up, he reported no pain, and there was no tenderness on examination. He was discharged from active care at that time. The clinical course is presented in Table 3.

**Table 3:** The timeline of the patient’s clinical course

Timeline (Months)	Event
0	First noticed symptoms
1-120	Seen by multiple orthopedic surgeons
129	Seen by a hand surgeon
130	Surgery by a hand surgeon
131	Discharged by a hand surgeon

## Discussion

Clinical presentation and the diagnostic triad

Three core clinical signs characterize typical glomus tumors: point tenderness, nail discoloration, and pain triggered by cold exposure or minor trauma [4]. This constellation of findings constitutes the classic clinical picture of a typical subungual glomus tumor. Corresponding to these three clinical signs are three diagnostic tests: the Love pin test, the Hildreth test, and the cold sensitivity test, whose reported sensitivities and specificities are summarized in Table 4. Despite these tests, there is still a significant delay in diagnosis. Unfortunately, the classic triad is often incomplete or entirely absent in extradigital tumors, making it harder to promptly diagnose [1].

**Table 4:** Reported sensitivity, specificity, accuracy, and predictive values of clinical diagnostic tests for glomus tumor, with MRI and transillumination included for comparative reference. MRI, magnetic resonance imaging; NPV, negative predictive value; NR, not reported; PPV, positive predictive value

Test	Study (Year)	n	Sensitivity	Specificity	Accuracy	PPV / NPV
Love Pin Test	Bhaskaranand and Navadgi, 2002 [2]	14	100%	NR	78%	NR
Love pin test	Netscher et al., 2012 [5]	18	100%	NR	NR	NR
Cold sensitivity test	Bhaskaranand and Navadgi, 2002 [2]	14	100%	100%	100%	NR
Cold sensitivity test	Netscher et al., 2012 [5]	18	100%	100%	100%	NR
Hildreth test	Giele, 2002 [4]	42	92%	91%	NR	PPV 92%
Hildreth test	Giele, 2002 [4]	42	92%	91%	NR	NPV 91%
Hildreth test	Lin et al., 2010 [6]	28	Lower	High	NR	NR
Transillumination	Cohen, 2023 [7]	–	23–38%	90%	NR	NR
MRI (for comparison)	Joseph and Posner, 1983 [8]	42	90%	50%	NR	PPV 97%
MRI (for comparison)	Joseph and Posner, 1983 [8]	42	90%	50%	NR	NPV 20%

Taken together, the data presented in Table 4 suggest that delayed diagnosis is more often related to under-recognition than deficiencies in available diagnostic tools. The Love pin test and cold sensitivity test each demonstrate 100% sensitivity across multiple independent series, a finding that is uncommon in clinical medicine. The Hildreth test, while more variable in its reported sensitivity (71.4% to 92%), consistently achieves 100% specificity in the studies that measure it, making a positive result essentially confirmatory [9,10]. Taken in combination, as Netscher et al. recommend, these three bedside tests constitute a diagnostic battery that requires no imaging, no specialist referral, and no more equipment than a pinhead and a tourniquet cuff. These findings suggest that delayed diagnosis may reflect a lack of clinical suspicion rather than a lack of available diagnostic tools.

The MRI data complicate this picture in an instructive way. Giele [4] reported a specificity of only 50% for MRI, meaning that half of lesions flagged by imaging in that series were not glomus tumors, alongside a negative predictive value of just 20%, indicating that a negative MRI provides very little reassurance. All four false-negative MRIs in that series were attributed to tumor size of 2-3 mm, well within the range of clinically symptomatic lesions. Ishii et al.'s more recent series (2026) places preoperative MRI diagnostic accuracy at 67%, consistent with this earlier finding [9]. These numbers are not an argument against imaging; on the contrary, Lin et al.'s landmark 75-case analysis found zero recurrence among patients who received preoperative MRI or ultrasound [6], a finding that speaks to the value of imaging in surgical planning even when it cannot guarantee a diagnosis. Instead, the MRI data underscore a specific danger: a negative scan in the face of a consistent clinical picture should never be used to dismiss the diagnosis. In the cases presented in this review, imaging was either not obtained, not interpreted in the context of the clinical triad, or falsely reassuring - a pattern that the literature suggests is neither unusual nor excusable.

The cold sensitivity test warrants particular attention in the context of extradigital presentation. While it achieves perfect sensitivity and specificity in digital glomus tumor series, its utility diminishes substantially when the tumor is extradigital: cold-induced exacerbation of pain is frequently absent in these cases, as Cohen noted [7], and its absence may remove glomus tumor from the differential entirely in the mind of a clinician already unfamiliar with the diagnosis. This creates a compounding effect: the test most likely to trigger clinical suspicion is precisely the test most likely to be negative in the presentation most likely to be missed. In the forearm case presented in this review, this dynamic is directly implicated in a diagnostic delay spanning a decade.

Diagnostic delay problem

The literature on glomus tumors is, in many respects, one of missed opportunities. Despite a clinical triad so characteristic that it approaches a pathognomonic presentation - localized point tenderness, paroxysmal pain, and cold sensitivity - these tumors have demonstrated a remarkable capacity to evade timely diagnosis, persisting undetected for years while patients accumulate specialist consultations, misdiagnoses, and, in some cases, psychiatric referrals. The numbers are striking in their consistency: across multiple large series, the mean interval between symptom onset and correct diagnosis has been reported to range from seven to nine years [10]. Ishii et al.'s more recent series of 11 patients reported a diagnostic delay of 8.6 ± 6.3 years regardless of tumor location [9]. At the extreme end of this spectrum sits a single reported case of a 40-year diagnostic delay - certainly an outlier but one that illuminates just how prolonged the window of misdiagnosis can become [11]. Therefore, the relevant question is not whether diagnostic delay is a problem in glomus tumor management. The more pressing question (and the one this review seeks to address) is whether that delay carries consequences beyond the suffering of the interim years, manifesting ultimately in the operating room as a higher burden of recurrence.

Having established the magnitude of diagnostic delay, it is important to examine the factors contributing to it. These tumors are often too small, too deeply situated, or located in atypical anatomical regions to be readily identified on clinical examination. According to Cohen, patients see an average of 2.5 specialists before a diagnosis is reached; these specialists include acupuncturists, dermatologists, neurologists, and chiropractors [7]. In some cases, patients are incorrectly given a psychiatric diagnosis when the lesion is too small or too deep to be detected [9]. In addition, the triad classically seen with subungual tumors is often absent when glomus tumors occur in extradigital locations [12]. This highlights the limitations of relying on classical clinical presentation when establishing a diagnosis.

Atypical presentations are not the only cases that are misdiagnosed - often a superficially plausible alternative diagnosis can mislead even experienced surgeons. In a small finger case, a glomus tumor was initially misdiagnosed as an ingrown nail, leading to a partial nail removal that only provided transient relief [13]. Collectively, the delays and misdiagnosis can create emotional, physical, and financial burdens for patients and the healthcare system.

Extradigital problem: a compounded challenge

Extradigital tumors warrant separate consideration because the mechanisms contributing to diagnostic delay are distinct and often more difficult to overcome. The classic triad is less reliable, the locations are novel, and physician awareness is even lower. In fact, only 20% of extradigital glomus tumors were correctly diagnosed by the initial treating physician [14]. Thus, approximately 80% of extradigital glomus tumors are misdiagnosed or experience delayed diagnosis at the initial presentation, leading to prolonged grievance for the patients involved. These extradigital tumors often lack the cold sensitivity classically shown by their subungual counterparts, removing perhaps the most intuitive clinical cue. In addition, deeper lesions may present without any visible or tactile signs on physical examination [1]. The absence of visible or palpable findings can lead these lesions to be mistaken for neuralgia, arthritis, or even psychosomatic pain. Imaging of these tumors continues to confound as well. One reported case required 15 MRI examinations before a definitive diagnosis was established [15]. This highlights the importance of precise site marking; even more crucial is clear communication between patient, physician, and radiologist.

Recurrence rates, satellite lesions, and preoperative imaging

Reported recurrence rates for glomus tumors vary considerably across studies. Most series report recurrence rates ranging from 12% to 18%, although rates as high as 33.3% have also been reported [10]. While this rate is higher than preferred, it may be reduced. An analysis of 75 cases reported a recurrence rate of 17%, but with zero recurrence in patients who received a preoperative MRI or ultrasound, highlighting the importance of thorough workup and planning [6]. In addition, skin-colored tumors and nail matrix locations are associated with a higher recurrence risk, emphasizing the trickiness of nontraditional presentation [6].

Oftentimes, what appears to be recurrence may possibly be a synchronous satellite lesion that matures to symptomatic size after the excision of the original lesion. This theory was first proposed in 1979 and has been supported by more recent studies. The recurrent lesion appeared in an anatomically distinct location from the original, but always in the same digit [16]. This pattern of spatial separation may support synchronous satellite lesions rather than merely an inadequate excision as the cause of recurrence. The timing of recurrence may also provide insight into its underlying cause; early recurrence can be suggestive of an incomplete excision, whereas a later recurrence (more than one year) may represent missed satellite lesions that then grew to symptomatic size [17].

One of the most compelling findings is the absence of recurrence among patients who underwent preoperative imaging. This may indicate a relationship between imaging and improved outcomes. If a patient goes years without a proper diagnosis, they are also going years without proper imaging, meaning the surgical planning is less precise, satellite lesions are more likely to be overlooked, and recurrence risk is higher. However, imaging findings should always be interpreted in conjunction with a complete physical examination. Preoperative MRI achieves accurate diagnosis in only six out of nine cases, meaning that clinical examination and imaging must be used in conjunction for better outcomes [18].

A table of the articles selected, listed by author, and study titles are listed below (Table 5).

**Table 5:** Literature review MP, metacarpophalangeal; MRI, magnetic resonance imaging

Article Reference/Link	Article Type/Focus	Recurrence	Digital/ Extra- digital)	Tumor Location	Delay	Diagnostic Method	Treatment	Outcome	Relevance to Review
Gombos and Zhang, 2008 [19]	Overview of glomus tumors	No	Digital and extradigital	Subungual (most common), also extradigital	Not applicable (review article)	Literature review	Surgical excision	Generally excellent	Background/overview on glomus tumor pathology, classification, and treatment
Kaufman et al., 2019 [10]	Unusual case of inner ear glomus tumor with recurrence and malignancy	Yes	Extradigital	Middle ear and mastoid	Not reported	Surgical excision + histology	Surgery; radiotherapy considered	Recurrence; malignant transformation	Establishes recurrence and rare malignant conversion; unusual extradigital site
Schiefer et al., 2006 [20]	Retrospective comparative study: clinical and pathological features of extradigital vs. digital glomus tumors	No (34/38 treated successfully without recurrence)	Extradigital (primary focus)	Upper extremities most common (n=25); wrist, forearm, arm	Not specified; misdiagnosis rate implies substantial delay	Clinical examination; histology; imaging in subset	Simple surgical excision	89.5% success rate without recurrence after excision	Critical statistic: only 20% of extradigital glomus tumors correctly diagnosed by initial physician; cold sensitivity often absent extradigitally; directly supports delayed diagnosis argument for forearm case
Joseph and Posner, 1983 [8]	Case series (3 cases): glomus tumors at the wrist	No	Extradigital	Wrist (ulnar aspect / dorsoulnar)	Up to 10 years (case 2: 10 years; case 1: 6 months post-injury onset)	Ethyl chloride cold spray provocative test; clinical examination; surgical + histology	Surgical excision under tourniquet with magnification	Complete pain relief in all 3 cases	Describes rare extradigital wrist location; ethyl chloride test useful for cold sensitivity diagnosis; supports delayed diagnosis theme
Gandhi et al., 2010 [21]	Retrospective case series: anatomic location of recurrences after excision	Yes (5/12 patients)	Digital	Subungual and periungual (distal phalanx)	Average 16.8 months pre-surgery in recurrence group	Clinical (pain, tenderness, cold sensitivity); MRI in 1 case; histology	Surgical excision; revision surgery for recurrences	5/12 recurrences; all recurred at new anatomic sites in same digit	Directly relevant: recurrences may reflect synchronous satellite lesions, not failed excision; supports careful surgical search; relevant to recurrence case
Andreoni et al., 2018 [1]	Case report: intravascular extradigital glomus tumor of forearm vein	No	Extradigital	Median antebrachial vein, volar proximal forearm	10 years	MRI (T2 hyperintense mass abutting vein); histology after excision	Complete surgical resection of venous segment	Complete resolution at 2-month follow-up	Directly parallels forearm case in review; 10-year diagnostic delay; intravascular location is very rare; extradigital presentation mimicked neuropathic pain
Lopes et al., 1997 [13]	Imaging rounds case report: subungual glomus tumor of small finger	No	Digital	Distal phalanx, radial periungual / subungual (small finger)	At least 1 year (initially misdiagnosed as ingrown nail)	MRI (T2 hyperintense, cortical interruption); histology; X-ray initially normal	Surgical excision	Complete relief; excellent outcome	Illustrates diagnostic delay and misdiagnosis (ingrown nail); MRI utility; classic subungual presentation; parallels typical case in review
Ishii et al., 2026 [9]	Retrospective case series: diagnostic delay and clinical characteristics; MRI utility in preoperative diagnosis	No (0/11 after complete excision)	Digital	Subungual (73%); non-subungual (27%)	Mean 8.6 ± 6.3 years (comparable across subungual and non-subungual)	Clinical examination (point tenderness most consistent finding, 91%); preoperative MRI (9/11); histology	Complete surgical excision in all cases	Complete symptom resolution in all; no recurrence	Strongest direct evidence for diagnostic delay argument; non-subungual tumors never correctly diagnosed preoperatively; MRI alone insufficient; supports point tenderness as key clinical clue
Lin et al., 2010 [6]	Retrospective analysis of factors associated with recurrence in 75 cases	Yes (13/75, 17%)	Digital	Finger (70 cases); toe (5 cases)	Mean 3.9 years	Clinical; preoperative MRI or ultrasound in subset	Surgical excision	17% recurrence; zero recurrence in patients who had preoperative MRI or ultrasound	KEY paper for delay → recurrence argument: preoperative imaging (enabled by timely diagnosis) associated with zero recurrence; skin-colored tumors and nail matrix location = higher recurrence risk
Newman et al., 2015 [22]	Case report: subungual glomus tumor with 10-year diagnostic delay; review of diagnostic pitfalls	Not reported (risk noted)	Digital	Subungual (right thumb)	10 years	Clinical; MRI; histology	Surgical excision	Resolution of symptoms	Cites well-documented mean 7-year diagnostic delay; links incomplete excision risk to poor margin definition; supports argument that delay → worse surgical planning → higher recurrence risk
Altarifi et al., 2024 [11]	Case report: subungual glomus tumor with extreme 40-year diagnostic delay	Not reported	Digital	Subungual	40 years (extreme outlier vs. documented mean of ~7 years)	Clinical triad; US and MRI; histopathology confirmatory	Surgical excision	Not reported	Demonstrates extreme end of diagnostic delay spectrum; reinforces that misdiagnosis is systemic and prolonged; useful for framing the urgency of clinical awareness
Lee et al., 2011 [18]	Retrospective cross-sectional institutional series: 31 cases over ~4 years	No (0/31)	Digital (majority)	Finger (61.3%); other sites	Mean 44.0 months (~3.7 years)	Clinical; histology; imaging in subset	Complete surgical excision	No recurrence despite involved margins in 3 cases	Large institutional series quantifying diagnostic delay (~44 months average); lump visible in <50% of cases, reinforcing occult nature; supports need for heightened clinical suspicion
Ooi et al., 2025 [16]	Case report: recurrent intramedullary glomus tumor; novel surgical approach	Yes	Digital	Distal phalanx (intramedullary)	2 years before initial surgery	Clinical; ultrasound (initially unremarkable); histology	Re-excision with transungual approach and cement spacer	Resolution after revision surgery	Supports satellite lesion theory: early recurrence (<1 year) = incomplete excision; late recurrence (>1 year) = missed satellite lesions; useful mechanistic framework for recurrence case
Giele, 2002 [4]	Prospective clinical study: sensitivity, specificity, and predictive values of Hildreth's test and MRI for glomus tumor diagnosis	Not reported	Digital	Hand (subungual, periungual)	Not reported	Hildreth's test; MRI; histology	Surgical excision	MRI: 90% sensitive, 50% specific, 97% PPV, 20% NPV; 4 false-negative MRIs all due to small tumor size (2–3 mm)	Key reference for clinical test accuracy: Hildreth's 92% sensitive, 91% specific; establishes that MRI can miss small tumors; supports argument that clinical tests are essential when imaging is negative
Serra et al., 1985 [23]	Case report: osseous glomus tumor at the MP joint articular surface	No	Digital (unusual osseous site)	Head of the third metacarpal bone (MP joint)	Not reported	Roentgenogram (osteolytic lesion) + histology after excision	Surgical excision with curettage; MP joint opened	Complete resolution; no recurrence	Rare primary osseous glomus tumor at atypical location; supports extradigital/extrasubungual presentation
Sahu et al., 2026 [17]	Case report: extradigital forearm glomus tumor with diagnostic delay; discussion of missed diagnosis pitfalls	Not reported	Extradigital	Forearm	Chronic (duration not specified); multiple prior workups negative	Clinical (localized tenderness, cold sensitivity); imaging; histology after excision	Surgical excision	Complete symptom resolution	Most directly parallel to forearm case in review; explicitly discusses causes of diagnostic delay in extradigital tumors; advocates systematic approach of clinical examination + imaging for early diagnosis; January 2026 (very recent)
AlNuaim et al., 2022 [12]	Case report: extradigital glomus tumor of the elbow with diagnostic delay; discussion of systemic causes of missed diagnosis	Not reported	Extradigital	Elbow (posterolateral)	3 years	Clinical; MRI; histology	Excisional biopsy (curative)	Complete resolution after excision	Explicitly discusses why extradigital tumors are delayed in diagnosis; classic triad often absent; patients visit multiple departments; highlights emotional, physical, and financial burden of delayed diagnosis on patients and healthcare system
Cohen, 2023 [7]	Case report + comprehensive literature review: extradigital wrist glomus tumor misdiagnosed as foreign body reaction; review of diagnostic pitfalls	No	Extradigital	Wrist (flexor surface)	Not specified; misdiagnosed as foreign body reaction prior to excision	Excisional biopsy (3-mm punch); histology confirmatory	Excisional biopsy	Complete pain resolution; no recurrence	Cold sensitivity absent → misdiagnosis; patients averaged 2.5 specialist consultations before diagnosis; psychiatric misdiagnosis assigned in some cases; spectrum of clinicians involved highlights systemic awareness gap

Limitations of this report should be considered. The patients’ long-term outcomes, recurrence rates, and symptom relief were unknown after they were discharged from active care. As a series of three case reports, the findings have limited generalizability. However, as it highlights the diagnostic challenges regarding glomus tumors, this report may guide diagnostic decision-making in these cases and facilitate the reduction of undesired outcomes due to delayed diagnosis and treatment. Another limitation is that patient-reported outcome measures were not obtained.

## Conclusions

Glomus tumors remain an important but frequently overlooked cause of chronic localized pain. The three presented cases demonstrate that delayed diagnosis can occur across both classic and atypical presentations, often resulting in years of unnecessary symptoms before definitive treatment. Surgical excision provided complete symptom resolution in each patient, emphasizing the excellent prognosis once these lesions are recognized. Greater recognition of these lesions, earlier incorporation of targeted imaging into surgical planning, and increased awareness of the possibility of satellite lesions may help shorten the diagnostic odyssey that characterizes this disease and improve patient outcomes. Although glomus tumors are uncommon, they should no longer remain hidden in plain sight.
